# Assessment of Sarcopenia and Obesity in Patients with Myasthenia Gravis Using Dual-Energy X-ray Absorptiometry: A Cross-Sectional Study

**DOI:** 10.3390/jpm11111139

**Published:** 2021-11-03

**Authors:** Che-Cheng Chang, Yen-Kung Chen, Hou-Chang Chiu, Jiann-Horng Yeh

**Affiliations:** 1 Department of Neurology, Fu Jen Catholic University Hospital, Fu Jen Catholic University, New Taipei City 24352, Taiwan; changcc75@gmail.com; 2 Ph.D. Program in Nutrition and Food Sciences, Human Ecology College, Fu Jen Catholic University, New Taipei City 24205, Taiwan; 3 Department of Nuclear Medicine and PET Center, Shin Kong Wu Ho-Su Memorial Hospital, Taipei 11101, Taiwan; M004149@ms.skh.org.tw; 4 School of Medicine, Fu Jen Catholic University, New Taipei City 24205, Taiwan; m001012.hc@gmail.com; 5 School of Medicine, Taipei Medical University, Taipei 11031, Taiwan; 6 Department of Neurology, Taipei Medical University, Shuang-Ho Hospital, New Taipei City 23561, Taiwan; 7 Department of Neurology, Shin Kong Wu Ho-Su Memorial Hospital, Taipei 11101, Taiwan; 8 Department of Neurology, Kaohsiung Medical University, Kaohsiung 80708, Taiwan

**Keywords:** sarcopenia, obesity, myasthenia gravis, dual-energy X-ray absorptiometry, neuromuscular disease, body composition

## Abstract

Sarcopenia and obesity can negatively impact quality of life and cause chronic fragility, and are associated with neuromuscular diseases, including myasthenia gravis (MG). The long-term consequences of body composition changes in chronic MG remain unknown; we therefore evaluated changes in body composition, including sarcopenia, obesity, lean body mass, and the prevalence of sarcopenic obesity in patients. In this cross-sectional study, 35 patients with MG (mean age: 56.1 years) and 175 matched controls were enrolled. Body fat mass and skeletal muscle mass were measured using whole body dual-energy X-ray absorptiometry. Patients with MG exhibited a higher prevalence of obesity and higher android adiposity and total body fat percentage than those of controls. Although the prevalence of sarcopenia and sarcopenic obesity did not increase with age, there was a decrease in arm and android muscle mass in patients with MG compared with controls. Lower muscle mass percentages were correlated with increased age and MG severity, but not with corticosteroid use. Thus, MG is associated with increased risk for obesity and decreased muscle mass with aging, regardless of corticosteroid use. Therefore, accurate diagnosis of body composition changes in MG could facilitate the application of appropriate therapies to promote health, improve quality of life, and prevent fragility.

## 1. Introduction

Myasthenia gravis (MG) is an autoimmune disorder affecting the postsynaptic muscle membrane of the neuromuscular junction (NMJ) that can cause fluctuation of muscle weakness in skeletal muscles. The extraocular muscles are the most frequently affected, usually asymmetrically, with symptoms of diplopia and ptosis. However, patients can also develop more diffuse weakness, including in the bulbar, limb, and respiratory muscles, which can lead to a critical condition requiring intensive care [1]. Treatment includes symptomatic therapy along with immunosuppressants [2,3]. These treatment strategies are very effective in improving muscle strength, functional abilities, quality of life, and survival rate [4]. However, some studies revealed that patients with MG exhibited low muscle mass and poor cardiovascular physical fitness despite effective medical control of the condition. In an animal study, muscle mass loss was observed in experimental autoimmune myasthenia gravis (EAMG) mice, causing chronic fatigue and muscle weight loss [5]. The long-term muscle status of patients with MG has not been fully elucidated.

Changes in body composition and loss of skeletal muscle mass are common features of the aging process [6]. Pathologic age-related conditions, including sarcopenia and obesity, can exacerbate these tendencies and cause decreased muscle mass, functional decline, increased cardiovascular risk, and disability; furthermore, they are associated with various comorbidities and metabolic abnormalities [7]. Sarcopenia and obesity have become important health issues in our aging society, with consequential functional decline, increased cardiovascular risk, and contribution to disability [8]. Sarcopenia can also be combined with obesity, a condition termed sarcopenic obesity, which can lead to a significant decline in quality of life and cause impairment of physical performance compared with sarcopenia or obesity alone [9,10].

Currently, there are several methods to assess body composition changes. The International Society for Clinical Densitometry recommends dual-energy X-ray absorptiometry (DXA) to accurately measure body composition [11]. DXA is a preferred method of evaluation because of its accuracy. Compared with computed tomography or magnetic resonance imaging, the advantages of DXA include low radiation, ease of use and a precise, consistent measurement that is unaffected by human factors. Using this method of measurement to determine body composition may provide a better understanding of the changes associated with MG [12,13].

Although sarcopenia develops mainly due to aging, it can also develop secondary to other etiologies, such as environmental causes, disease, inflammatory reactions, mitochondrial abnormalities, hormonal changes, and loss of NMJ stability, including some neuromuscular diseases [14,15]. MG and sarcopenia share similar characteristics regarding the disruption of the NMJ function, leading to muscle weakness and loss of muscle mass [16]. A retrospective cohort study in Taiwan demonstrated that MG is associated with an increased risk of osteoporosis [17]. Due to continual changes in the course of the disease and consequent medication regimens, patients with MG may develop body composition changes, including sarcopenia and obesity, which have both been associated with poorer health conditions and adverse outcomes. While these secondary effects aggravate adverse outcomes in MG, the underlying changes in body composition have received little attention in the literature. Consequently, this study aimed to document the changes in body composition, including muscle mass, fat distribution, obesity, and sarcopenia, in patients with MG.

## 2. Materials and Methods

### 2.1. Participants and Study Design

This cross-sectional study included patients with MG who were followed up at the Neurology Outpatient Clinic of the Shin Kong Wu Ho-Su Memorial Hospital, Taiwan during 2018 and had undergone whole body dual-energy X-ray absorptiometry (DXA). In 2018, age- and sex-matched subjects were identified from the Database of Health Examination in Shin Kong Wu Ho-Su Memorial Hospital and recruited as the control group.

The inclusion criteria for patients with MG were (1) Myasthenia Gravis Foundation of America (MGFA) classes II and III, and (2) no medication adjustment in the previous 6 months. The exclusion criteria were (1) unstable MG symptoms, and (2) history of intensive immuno-modulation therapy, including immunoglobulins, high dose intravenous corticosteroid, or plasmapheresis during the 6-month period preceding enrollment, because use of these short action immunotherapies indicates that the patient has a life-threatening condition with recent unstable symptoms. Patients were eligible if they were diagnosed with MG based on the MGFA criteria [18]. Briefly, the diagnosis of MG was based on fluctuating muscle weakness with fatigability, decreased symptom severity after use of acetylcholinesterase inhibitors, decremental changes in repetitive nerve stimuli on repetitive nerve stimulation test, or presence of anti-acetylcholine receptor (AchR) autoantibodies [18].

This study complied with the principles of the Declaration of Helsinki and was approved by the ethical committee of Shin Kong Wu Ho-Su Memorial Hospital (No. 20170914R and No. 20200903R). All participants in the MG group provided written informed consent before being enrolled in the study; however, since the control group’s data were used retrospectively, informed consent for this group was waived by the ethics committee.

### 2.2. Data Collection and Clinical Measurement

Information on the patients’ medical history was collected at the time of evaluation, including the average daily dose of corticosteroids and all MG-related medications. The clinical status and MG severity were determined based on the recommendations of the MGFA [18]. Trained researchers assessed the quantitative MG (QMG) and MG quality of life (MG-QOL) scores according to previous studies [19,20]. Body mass index (BMI) was calculated as the body weight (kg) divided by the height squared (m^2^). The daily doses of prednisone and other immunosuppressants were extracted from the medical records.

### 2.3. Body Composition Assessment

Body composition assessment was performed using DXA by certified radiological technologists. Images were obtained with patients in the supine position and were analyzed using the manufacturer’s specifications and normative data. Using the DXA results, we evaluated the following parameters: appendicular (arms and legs) fat mass (kg); appendicular lean muscle mass (kg); arm, leg, appendicular, android, gynoid, and whole body adiposity (%); arm, leg, appendicular, android, gynoid, and whole body lean muscle mass percentage (%); appendicular skeletal muscle mass (ASM, kg). The ASM index (ASMI) was calculated by dividing the ASM (fat-free mass in the arms and legs; kg) by the height squared (m^2^). The android-to-gynoid (A/G) ratio was calculated as the ratio of android adiposity to gynoid adiposity.

### 2.4. Definition of Sarcopenia, Obesity, and Sarcopenic Obesity

Based on the DXA data, sarcopenia was defined as an ASMI < 7.0 kg/m^2^ in men and 5.4 kg/m^2^ in women, according to the criteria for Asians [21,22]. Obesity was defined if one of these four conditions were met: high A/G ratio (>0.80 in men, >0.62 in women), high android fat mass (>2.16 kg in men, >1.95 kg in women), high body fat percentage (>31.8% in men, >38.8% in women), or BMI > 25 kg/m^2^, according to previous cohort studies in Asians [23]. Sarcopenic obesity was defined if both the criteria for obesity and sarcopenia were fulfilled.

### 2.5. Statistical Analysis

There were substantial differences in age and sex between the MG and non-MG (control) groups; therefore, we adopted frequency matching using age (age groups: 40–49, 50–59, and 60–70 years), and sex. Each patient in the MG group was age- and sex-matched with five controls from the non-MG group. In the stratification analysis by age group, matching was repeated three times. Likewise, when stratifying the patients with MG based on steroid use, matching was repeated twice.

The clinical features were compared between groups (i.e., MG vs. control) using Fisher’s exact test for categorical variables or the independent sample *t*-test for continuous variables. The body composition was compared between the MG group and the matched control group using the generalized estimating equation, which accounted for the outcome dependency within the same matching pair by using robust standard error and exchangeable working correlation. Comparison of the clinical features and body compositions between subgroups (i.e., obesity vs. non-obesity; steroid use vs. non-steroid use) was performed using Fisher’s exact test for categorical variables or the independent sample *t*-test for continuous variables. All tests were two-tailed, and *p* < 0.05 was considered statistically significant. No adjustment of multiple testing (multiplicity) was made in this study. Data analysis was performed using IBM SPSS Statistics for Windows, version 25 (IBM Corp., Armonk, NY, USA).

## 3. Results

### 3.1. Clinical Features of Subjects

Thirty-five patients with MG, including 22 women, and 175 age- and sex-matched controls were included in this study. The clinical characteristics of the MG and matched control groups are shown in Table 1. The mean age in the MG group was 56.1 ± 8.6 years. Twenty-one patients with MG (60%) had received steroids within 6 months with a mean duration of corticosteroid use of 7.0 ± 5.3 years and an average daily dose of 5.3 ± 5.7 mg (0.1 ± 0.1 mg/kg). The average disease duration was 12.3 ± 10.6 years. All patients were positive for AChR autoantibodies. Ten patients (28.6%) had received other immunosuppressant treatment including azathioprine and Mycophenolate Mofetil. Some patients with MG had comorbidities, including hypertension (*n* = 5, 14.2%), diabetes (*n* = 2, 5.7%), hyperlipidemia (*n* = 3, 12%), and cardiovascular disease (*n* = 3, 12%). Three patients reported the co-occurrence of autoimmune diseases (ankylosing spondylitis, rheumatoid arthritis, and systemic lupus erythematosus).

According to the DXA results, the prevalence of obesity was significantly higher in the MG group than in the control group (40% vs. 1.1%, *p* < 0.001). Notably, the prevalence of sarcopenia was not significantly different between the groups. Only one patient with MG (2.9%) had sarcopenic obesity compared with none in the control group (2.9% vs. 0%, *p* = 0.167).

### 3.2. Comparison of Body Composition between the MG and Non-MG Groups

**Figure 1:**
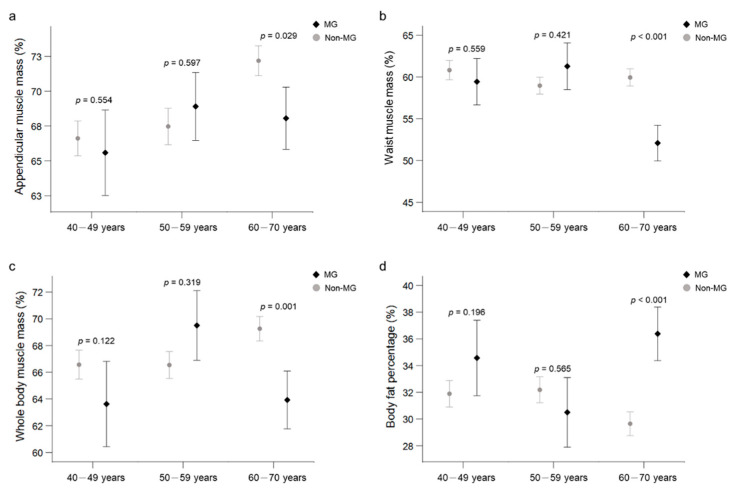
Trend toward lower body muscle mass percentages and higher body fat adiposity in the MG group, particularly regarding appendicular (**a**), waist (**b**), whole body (**c**) muscle mass percentages and body fat percentage (**d**) in the older subgroup. MG, myasthenia gravis group; Non-MG, control groups.

DXA-derived body composition measures in patients with MG and matched control subjects are summarized in Table 2. Patients with MG had lower arms (regression coefficient (B), −3.11%; 95% confidence interval (CI), −6.01 to −0.21%) and waist (B, −3.10%; 95% CI, −6.15 to −0.06%) muscle percentages but higher body fat percentage (B, 3.17%; 95% CI, 0.48 to 5.85%) and higher A/G ratio (B, 0.59; 95% CI, 0.53 to 0.65) than the controls. After stratification by age (40–49, 50–59, and 60–70 years), a trend toward lower body muscle mass percentages with increasing age was observed in the MG group, particularly in the appendicular, waist, and whole body measurements in the 60–70 year subgroup (Figure 1, Table 3, and Supporting Information, Tables S1 and S2).

### 3.3. Subgroup Analysis of the MG Group

A subgroup analysis was performed on the MG group based on the presence or absence of sarcopenia and obesity, type of MG, QMG score, and disease duration (Supporting Information, Tables S3 and S4). No significant differences were observed in the QMG scores, MG-QOL scores, disease duration, and daily steroid doses between the sarcopenia and non-sarcopenia or the obesity and non-obesity subgroups. Patients with MGFA class III MG exhibited lower appendicular, gynoid, and whole body muscle mass percentages (Supporting Information, Table S3). There were no significant differences in any of the parameters between the low and high QMG score subgroups. Few differences were observed between the short and long disease duration subgroups (Supporting Information, Table S4).

### 3.4. Subgroup Analysis of the MG Group Based on Steroid Use

Further subgroup analysis was conducted on the MG group based on the use of steroids (Table 4 and Supporting Information, Tables S5–S7). In the subgroup of patients with MG treated with corticosteroids (n = 21, 60%), the average dose was 8.9 ± 4.7 mg per day (0.2 ± 0.1 mg/kg per day). The clinical characteristics and body composition of the subgroups of patients with MG (with or without steroid use) are presented in Table S5. Notably, despite the increasing A/G ratio and more prevalent obesity in both subgroups compared to the control groups (Table 4 and Supporting Information, Tables S6 and S7), there were no significant differences in body composition, A/G ratio, sarcopenia, or obesity prevalence between the subgroups (Table S5). However, after adjusting for sex, age, disease duration and MGFA (type of MG), the results of multivariable linear regression analysis demonstrated that the use of steroids was associated with a lower muscle mass in legs (regression coefficient −1.87, 95% confidence interval (CI) −3.13 to −0.62) and appendicular (regression coefficient −2.25, 95% CI, −4.01 to −0.49) (Table S8). When patients with MG were categorized into subgroups based on steroid use, there were some differences in body composition between the MG and control groups but no differences in muscle mass percentage and frequency of sarcopenia (Supporting Information, Tables S5–S7).

## 4. Discussion

In this study, we evaluated the body composition changes in patients with MG of different ages and disease severity, compared with those in healthy control subjects. Our results demonstrated a higher prevalence of obesity in patients with MG than in controls. Moreover, patients with MG had higher body fat percentages and android body adiposity compared with controls. Although the prevalence of sarcopenia was not increased in the MG group, there was a decrease in arm and android muscle mass compared with those of the control group. Interestingly, a trend of lower body muscle mass percentages was correlated with increasing age and MG severity but not with corticosteroid medication use. The higher the MGFA class, the lower the appendicular, gynoid, and whole-body muscle mass percentages. We observed body composition changes in whole body muscle mass percentages, particularly in the upper limbs, across the MG age groups. An increase in the prevalence of sarcopenic obesity was also noticed in patients with MG, although there was no significant difference compared with the control group.

The observed body composition changes in patients with MG, a higher prevalence of obesity and lower muscle mass, may be due to several factors, including the disease course and the decrease in physical activity because of muscle fatigability [1]. Previous studies reported that high corticosteroid accumulation may also cause increased prevalence of obesity and higher body fat in patients with MG. This difference in muscle loss may result from the disease course because long-standing disease may cause skeletal muscle wasting in MG [24]. Braz et al. demonstrated that a high cumulative corticosteroid dose in patients with MG could cause increased body fat and decreased lean body mass [25]. Corticosteroid use has several side effects, including weight gain, central obesity, and increased adiposity, and long-term corticosteroid use by patients with MG can also result in metabolic consequences, such as insulin resistance and diabetes [26]. However, in contrast to previous results, our study showed that body composition changes in fat adiposity and muscle mass were not associated with corticosteroid use but were possibly related to the disease severity and progression.

Fatigable limb weakness is the clinical hallmark of patients with MG, and this weakness is more prominent in the upper than in the lower limbs [27]. We confirmed that patients with MG had lower arm muscle mass percentages than those of the control group, which is in agreement with both clinical observation and previous reviews. A trend toward lower body muscle mass percentages in the older adult MG group was observed in the appendicular muscles. To our knowledge, no previous study has reported evidence of decreased muscle mass in the appendicular muscles in the disease course of anti-AchR-positive MG.

In this study, we identified decreases in muscle mass percentages in various parts of the body in patients with MG. Oosterhuis et al. reported muscle atrophy in patients with MG, and the atrophic muscles exhibited pathological neurogenic changes. Muscle mass loss or wasting is a rare finding that occurs in approximately 10% of these patients [24]. Farrugia et al. also reported facial and bulbar muscle wasting in patients with anti-AchR-positive MG in magnetic resonance imaging (MRI) studies [28]. Muscle atrophy in the face and tongue has been reported in patients with anti-muscle-specific kinase (anti-MuSK antibodies) in several studies [29,30,31] which can cause prominent bulbar palsy compared with anti-AchR-positive MG [32,33]. Early atrophy in a patient with MG detected using MRI before glucocorticoid use has also been reported [34].

In our study, muscle mass increased more prominently in the 50–59 year group. According to previous studies, muscle mass loss can begin from middle-age and decrease by ~1% per year, which can lead to a loss of >50% by the age of >80 years [35]. The increase in muscle mass in our study could be due to: (1) the smaller sample size, and (2) recent data reported that some factors had critical roles in insulin resistance that cause muscle mass loss, including the use of hormones, physical exercise, and nutrition status [36]. Our study lacks information regarding nutrition state and physical exercise. Collecting further multi-center cohort data, and recording the nutrition and physical exercise status of the participants to evaluate our findings could be important for future research.

The mechanism underlying muscle mass loss in patients with MG remains unclear. The EAMG models provided evidence of muscle atrophy due to blocked neuromuscular transmission molecules [5]. NMJs are important in maintaining normal muscle function and mass. Recent studies have reported age-associated changes that could precede the denervation of muscle fibers in NMJs, which play a significant role in the pathogenesis of sarcopenia [15,37,38]. Aging muscle in animals exhibited loss of postsynaptic clustering of AChRs compared with younger muscles [39]. Remodeling at the synapse of muscles and nerves plays important roles in aged muscles. Mice with MG and MuSK antibodies displayed dysfunction in the maintenance of NMJs, and an excessive degree of ramification and branching in atrophic muscles due to compensation for the breakdown of normal neuromuscular interactions [40], which eventually causes a decrease in muscle mass or atrophy [16].

The etiology of muscle wasting includes several possible factors, including (1) an NMJ transmission deficit that leads to muscle mass loss [24]; (2) long clinical course refractory to conventional therapy [41]; (3) reaction between antibodies and receptors; (4) chronic corticosteroid exposure, which was previously shown to cause an increase in adiponectin levels [25]. In our study, the muscle mass in patients with MG was significantly lower than that in the control group, which substantiates previous findings and indicates that the etiology may be due to the long clinical course and disease progression rather than being related to steroid use.

Understanding the composition changes in body fat and mass can aid in developing a new approach for health promotion and medical management of MG. One case report showed that muscle atrophy in patients with MG improved after immunotherapy, indicating that interruption of the immune response in the NMJ can delay neurogenic muscle atrophy [42]. Currently, several pharmacological agents that have the potential to treat sarcopenia are being investigated to enhance the management of MG, including tirasemtiv, a skeletal troponin activator that affects the NMJ disease [43]. Moreover, some studies have emphasized that the decrease in muscle mass can interfere with the treatment response and lead to limited recovery of muscle weakness. A case series that included ten patients with MG with prominent muscle atrophy reported improvement in the muscle atrophy after medical therapy [44]. Therefore, timely diagnosis of muscle mass loss and body composition changes in patients with MG may enable the application of appropriate therapies that are effective in preventing muscle wasting and improving patients’ daily function and muscle strength.

Our study was subject to the following limitations. First, the sample size of patients with MG was small, and the results cannot be considered to be representative of all patients with MG. Second, the total cumulative dose of corticosteroids was not evaluated. However, steroid dose could affect the body composition, and corticosteroids could cause sarcopenia or obesity. Third, we did not record the comorbidities, concurrent medications, and endocrine abnormalities (e.g., blood glucose, insulin levels, leptin and lipid profiles). Finally, we did not have any information regarding the physical activity, nutritional status, and dietary habits of the study participants. Therefore, future studies should involve larger cohorts and further evaluate the relationship between corticosteroid dose, nutrition state, and blood and body composition of MG patients, as well as the influence of physical activities on body composition, cardiovascular fitness, and lung function.

## 5. Conclusions

In summary, this study demonstrates that MG is associated with an increased risk of obesity and decreased muscle mass with aging, regardless of corticosteroid use. The observed changes may be related to the disease severity and progression. Screening patients with MG at risk for obesity and muscle mass loss, and early prophylactic intervention could improve their quality of life and prevent chronic fragility. Therefore, accurate diagnosis of body composition changes in MG, including loss of muscle mass and increased adiposity, may be important to facilitate the application of appropriate therapies to mitigate consequential complications and enhance the quality of life of patients.

## Figures and Tables

**Table 1:** Clinical characteristics of patients with MG and sex- and age-matched controls.

Variable	MG (*n* = 35)	Non-MG (*n* = 175)	*p*-Value
Male sex (%)	13 (37.1)	65 (37.1)	1.000
Age (years)	56.1 ± 8.6	56.0 ± 8.5	0.939
Age group (years)			1.000
40–49	10 (28.6)	50 (28.6)	
50–59	10 (28.6)	50 (28.6)	
60–70	15 (42.9)	75 (42.9)	
Obesity (%)	14 (40.0)	2 (1.1)	<0.001 *
Sarcopenia (%)	8 (22.9)	67 (38.3)	0.121
Sarcopenic obesity (%)	1 (2.9)	0 (0.0)	0.167
Medication for MG		-	-
Pyridostigmine	33 (94.2)		
Corticosteroid (CS)	21 (60.0)		
CS daily dose in last 6 months (mg)	5.3 ± 5.7		
CS daily dose (mg/kg)	0.1 ± 0.1		
Duration of CS exposure (yr)	7.0 ± 5.3		
Treat with immunosuppressants	10 (28.6)		
AChR-autoantibody positivity (%)	35 (100)		
Comorbid disease (%)			
Diabetes	2 (5.7)		
Hypertension	5 (14.2)		
Hyperlipidemia	3 (12.0)		
Cardiovascular disease	3 (12.0)		
Autoimmune disease	3 (12.0)		

* *p* < 0.05, MG, myasthenia gravis; CS, corticosteroid.

**Table 2:** Body composition in patients with MG and sex- and age-matched controls.

Variable	MG (*n* = 35)	Non-MG (*n* = 175)	Mean Difference (95% CI) ^a^	*p*-Value
Body mass index (kg/m^2^)	24.8 ± 4.6	23.6 ± 3.7	1.17 (−0.19, 2.54)	0.091
Fat mass (kg)				
Arms	2.1 ± 0.9	1.7 ± 0.6	0.38 (0.06, 0.69)	0.018 *
Legs	6.7 ± 2.7	5.9 ± 2.7	0.79 (−0.13, 1.72)	0.092
Appendicular	8.8 ± 3.4	7.6 ± 3.1	1.17 (−0.03, 2.37)	0.055
Muscle mass (kg)				
Arms	4.3 ± 1.5	4.2 ± 1.3	0.05 (−0.28, 0.39)	0.751
Legs	13.6 ± 3.1	13.0 ± 3.0	0.69 (0.01, 1.38)	0.048 *
Appendicular	17.9 ± 4.4	17.2 ± 4.2	0.75 (−0.22, 1.72)	0.131
Fat adiposity (%)				
Android	43.2 ± 9.3	40.1 ± 8.2	3.10 (0.06, 6.15)	0.046 *
Gynoid	39.9 ± 7.5	39.1 ± 9.1	0.81 (−1.34, 2.96)	0.460
Muscle mass (%)				
Arms	67.7 ± 10.7	70.9 ± 9.5	−3.11 (−6.01, −0.21)	0.035 *
Legs	67.6 ± 8.4	68.8 ± 9.7	−1.20 (−3.82, 1.42)	0.370
Appendicular	67.6 ± 8.6	69.3 ± 9.5	−1.66 (−4.19, 0.87)	0.199
Android	56.8 ± 9.3	59.9 ± 8.2	−3.10 (−6.15, −0.06)	0.046 *
Gynoid	60.1 ± 7.5	60.9 ± 9.1	−0.81 (−2.96, 1.34)	0.460
Whole body	65.4 ± 9.0	67.7 ± 7.7	−2.28 (−4.97, 0.40)	0.096
Android/gynoid fat ratio	1.1 ± 0.2	0.5 ± 0.2	0.59 (0.53, 0.65)	<0.001 *
Body fat percentage (%)	34.2 ± 8.4	31.0 ± 7.3	3.17 (0.48, 5.85)	0.021 *
ASMI (kg/m^2^)	6.7 ± 1.2	6.5 ± 1.1	0.18 (−0.07, 0.43)	0.168

* *p* < 0.05, ^a^ estimated using the generalized estimating equation. MG, myasthenia gravis; CI, confidence interval; ASMI, appendicular skeletal muscle mass index.

**Table 3:** Body composition in MG and sex- and age-matched controls aged 60–70 years.

Variable	MG (*n* = 15)	Non-MG (*n* = 75)	Mean Difference (95% CI) ^a^	*p*-Value
Body mass index (kg/m^2^)	25.8 ± 3.5	24.2 ± 3.2	1.57 (−0.01–3.16)	0.052
Fat mass (kg)				
Arms	2.3 ± 0.9	1.6 ± 0.6	0.64 (0.19–1.08)	0.005 *
Legs	6.5 ± 2.6	5.2 ± 2.0	1.28 (−0.22–2.78)	0.096
Appendicular	8.8 ± 3.4	6.9 ± 2.5	1.91 (0.02–3.80)	0.047 *
Muscle mass (kg)				
Arms	4.5 ± 1.5	4.5 ± 1.3	−0.01 (−0.42–0.41)	0.978
Legs	14.0 ± 3.3	13.6 ± 3.2	0.45 (−0.66–1.56)	0.431
Appendicular	18.5 ± 4.7	18.1 ± 4.4	0.44 (−1.04–1.92)	0.560
Fat adiposity (%)				
Android	47.9 ± 8.3	40.1 ± 8.8	7.86 (3.94–11.78)	<0.001 *
Gynoid	39.0 ± 8.2	35.9 ± 9.2	3.11 (−0.11–6.32)	0.058
Muscle mass (%)				
Arms	66.4 ± 10.4	72.8 ± 9.8	−6.41 (−9.39–−3.42)	<0.001 *
Legs	68.7 ± 8.6	72.0 ± 9.4	−3.34 (−7.63–0.95)	0.127
Appendicular	68.1 ± 8.6	72.2 ± 9.3	−4.14 (−7.85–−0.42)	0.029 *
Android	52.1 ± 8.3	59.9 ± 8.8	−7.86 (−11.78–−3.94)	<0.001 *
Gynoid	61.0 ± 8.2	64.1 ± 9.2	−3.11 (−6.32–0.11)	0.058
Whole body	63.9 ± 8.4	69.3 ± 8.0	−5.33 (−8.62–−2.04)	0.001 *
Android/gynoid fat ratio	1.3 ± 0.2	0.6 ± 0.2	0.68 (0.57–0.78)	<0.001 *
Body fat percentage (%)	36.4 ± 7.8	29.7 ± 7.7	6.73 (3.58–9.88)	<0.001 *
ASMI (kg/m^2^)	6.8 ± 1.3	6.8 ± 1.2	−0.07 (−0.41–0.27)	0.680

* *p* < 0.05, ^a^ estimated using the generalized estimating equation. MG, myasthenia gravis; CI, confidence interval; ASMI, appendicular skeletal muscle mass index

**Table 4:** Clinical characteristics of patients with MG and sex- and age-matched controls according to steroid use.

Variable	MG with Steroids (*n* = 21)	Non-MG (*n* = 105)	*p*-Value	MG without Steroids (*n* = 14)	Non-MG (*n* = 70)	*p*-Value
Male sex, no. (%)	9 (42.9)	45 (42.9)	1.000	4 (28.6)	20 (28.6)	1.000
Age (years)	57.1 ± 8.6	57.0 ± 8.4	0.948	54.7 ± 8.7	54.7 ± 8.5	0.986
Age group (years)						
40–49	5 (23.8)	25 (23.8)	1.000	5 (35.7)	25 (35.7)	1.000
50–59	5 (23.8)	25 (23.8)	1.000	5 (35.7)	25 (35.7)	1.000
60–70	11 (52.4)	55 (52.4)	1.000	4 (28.6)	20 (28.6)	1.000
Obesity	9 (42.9)	0 (0.0)	<0.001 *	5 (35.7)	0 (0.0)	<0.001 *
Sarcopenia	5 (23.8)	42 (40.0)	0.218	3 (23.1)	27 (38.6)	0.359
CS daily dose (mg/kg)	0.2 ± 0.1	-	-	-	-	-
Duration of CS (yr)	7.0 ± 5.3	-	-	-	-	-
Prednisolone daily dose during the previous 6 months (mg)	8.9 ± 4.7	-	-	-	-	-
Immune medication used	7 (33.3)	-	-	3 (21.4)	-	-
Disease duration (years)	15.1 ± 12.4	-	-	8.1 ± 4.6	-	-

* p < 0.05, Data are presented as numbers (percentages) or means ± standard deviations. MG, myasthenia gravis. CS, corticosteroid.

## Data Availability

All data supporting our conclusions are contained within the article.

## Supplementary Materials

The following are available online at https://www.mdpi.com/article/10.3390/jpm11111139/s1, Table S1. Body composition in patients with MG and sex- and age-matched controls aged 40–49 years, Table S2. Body composition in patients with MG and sex- and age-matched controls aged 50–59 years, Table S3. Clinical features and body composition of patients with MG according to the MG type, Table S4. Clinical features and body composition of patients with MG according to disease duration, Table S5. Clinical features and body composition of patients with MG according to steroid use, Table S6. Clinical features of patients with MG who received steroids and sex- and age-matched controls, Table S7. Body composition of patients with MG not treated with steroids and sex- and age-matched controls, Table S8. Multivariable linear regression for association of body composition and steroid use (with versus without)
