# Supplementary material for: Assessment of Sarcopenia and Obesity in Patients with Myasthenia Gravis Using Dual-Energy X-ray Absorptiometry: A Cross-Sectional Study
Source: J Pers Med. 2021 Nov 3;11(11):1139. doi: 10.3390/jpm11111139 (PMC8623024; doi:10.3390/jpm11111139)
Supplement: Supplementary file 1 [file jpm-11-01139-s001.zip › jpm-1409594-supplementary.pdf]

Supplemental Material

Table S1. Body composition in MG and sex- and age-matched controls aged 40–49 years

Table S2. Body composition in MG and sex- and age-matched controls aged 50–59 years

Table S3. Clinical features and body composition of MG according to the MG type

Table S4. Clinical features and body composition of MG according to disease duration

Table S5. Clinical features and body composition of patients with MG according to steroid use

Table S6. Clinical features of MG who received steroids and sex- and age-matched controls

Table S7. Body composition of MG not receive steroids and sex- and age-matched controls

Table S8. Multivariable linear regression for association of body composition and steroid use (with versus without)

Table S1. Body composition in MG and sex- and age-matched controls aged 40–49 years

Variable	MG (n = 10)	Non-MG (n = 50)	Mean difference (95% CI) [a]	*p*-value
Body mass index (kg/m2)	24.8 ± 6.6	22.9 ± 4.7	1.90 (-1.29–5.08)	0.244
Fat mass (kg)				
Arms	1.8 ± 0.8	1.6 ± 0.5	0.18 (-0.34–0.70)	0.507
Legs	7.7 ± 3.4	6.7 ± 3.7	1.05 (-0.46–2.56)	0.174
Appendicular	9.5 ± 4.0	8.3 ± 3.9	1.22 (-0.76–3.21)	0.228
Muscle mass (kg)				
Arms	4.0 ± 1.9	3.8 ± 1.2	0.15 (-0.72–1.03)	0.736
Legs	13.6 ± 3.4	12.4 ± 2.7	1.24 (-0.23–2.71)	0.099
Appendicular	17.6 ± 5.0	16.2 ± 3.5	1.39 (-0.85–3.63)	0.224
Fat adiposity (%)				
Android	40.6 ± 8.8	39.2 ± 8.2	1.39 (-3.27–6.05)	0.559
Gynoid	42.6 ± 7.6	41.9 ± 8.2	0.69 (-1.95–3.33)	0.608
Muscle mass (%)				
Arms	68.1 ± 12.0	69.5 ± 9.0	-1.36 (-6.41–3.69)	0.597
Legs	64.8 ± 9.4	65.8 ± 8.9	-1.06 (-4.35–2.22)	0.526
Appendicular	65.6 ± 9.7	66.6 ± 8.9	-1.03 (-4.45–2.39)	0.554
Android	59.4 ± 8.8	60.8 ± 8.2	-1.39 (-6.05–3.27)	0.559
Gynoid	57.4 ± 7.6	58.1 ± 8.2	-0.69 (-3.33–1.95)	0.608
Whole body	63.6 ± 10.1	66.6 ± 7.7	-2.95 (-6.68–0.79)	0.122
Android/gynoid fat ratio	1.0 ± 0.1	0.4 ± 0.1	0.54 (0.47–0.61)	<0.001*
Body fat percentage (%)	34.6 ± 8.9	31.9 ± 7.1	2.68 (-1.38–6.73)	0.196
ASMI	6.6 ± 1.4	6.1 ± 1.0	0.52 (-0.08–1.12)	0.088

MG, myasthenia gravis; CI, confidence interval; ASMI, appendicular skeletal muscle mass index. [a] estimated using the generalized estimating equation. * $p<0.05$

Table S2. Body composition in MG and sex- and age-matched controls aged 50–59 years

Variable	MG (n = 10)	Non-MG (n = 50)	Mean difference (95% CI) [a]	*p*-value
Body mass index (kg/m2)	23.4 ± 3.5	23.5 ± 3.2	-0.15 (-2.58–2.29)	0.906
Fat mass (kg)				
Arms	2.0 ± 0.9	1.8 ± 0.6	0.19 (-0.45–0.83)	0.565
Legs	6.0 ± 1.9	6.2 ± 2.2	-0.19 (-1.78–1.41)	0.818
Appendicular	8.0 ± 2.8	8.0 ± 2.7	0.002 (-2.17–2.18)	0.999
Muscle mass (kg)				
Arms	4.1 ± 0.8	4.1 ± 1.2	0.05 (-0.43–0.53)	0.841
Legs	13.1 ± 2.7	12.6 ± 3.0	0.52 (-0.30–1.34)	0.216
Appendicular	17.2 ± 3.4	16.7 ± 4.1	0.57 (-0.60–1.74)	0.343
Fat adiposity (%)				
Android	38.7 ± 8.8	41.0 ± 7.1	-2.31 (-7.94–3.32)	0.421
Gynoid	38.5 ± 6.4	41.1 ± 8.5	-2.52 (-6.88–1.85)	0.259
Muscle mass (%)				
Arms	69.4 ± 10.6	69.3 ± 9.0	0.09 (-6.69–6.86)	0.980
Legs	68.9 ± 7.2	67.0 ± 9.6	1.88 (-3.17–6.93)	0.465
Appendicular	68.9 ± 7.7	67.5 ± 9.3	1.44 (-3.88–6.76)	0.597
Android	61.3 ± 8.8	59.0 ± 7.1	2.31 (-3.32–7.94)	0.421
Gynoid	61.5 ± 6.4	58.9 ± 8.5	2.52 (-1.85–6.88)	0.259
Whole body	69.5 ± 8.2	66.5 ± 7.1	2.96 (-2.86–8.78)	0.319
Android/gynoid fat ratio	1.0 ± 0.2	0.5 ± 0.2	0.51 (0.43–0.60)	<0.001*
Body fat percentage (%)	30.5 ± 8.2	32.2 ± 6.8	-1.69 (-7.43–4.06)	0.565
ASMI	6.6 ± 0.9	6.3 ± 1.0	0.21 (-0.08–0.49)	0.156

MG, myasthenia gravis; CI, confidence interval; ASMI, appendicular skeletal muscle mass index. [a] estimated using the generalized estimating equation. * p<0.05

Table S3. Clinical features and body composition of MG according to the MG type

Variable	Type 2 MG (n = 21)	Type 3 MG (n = 14)	*p*-value
Male sex	10 (47.6)	3 (21.4)	0.162
Age (yr)	55.7 ± 8.9	56.9 ± 8.3	0.694
Age group (yr)			0.905
40–49	6 (28.6)	4 (28.6)	
50–59	7 (33.3)	3 (21.4)	
60–70	8 (38.1)	7 (50.0)	
Obesity	7 (33.3)	7 (50.0)	0.483
Sarcopenia	5 (23.8)	3 (21.4)	1.000
Body mass index (kg/m2)	24.1 ± 3.4	25.9 ± 5.8	0.252
Fat mass (kg)			
Arms	1.8 ± 0.8	2.4 ± 0.9	0.046*
Legs	6.1 ± 2.5	7.6 ± 2.8	0.092
Appendicular	7.9 ± 3.1	10.1 ± 3.4	0.061
Muscle mass (kg)			
Arms	4.3 ± 1.1	4.2 ± 1.9	0.759
Legs	13.8 ± 2.6	13.5 ± 3.8	0.809
Appendicular	18.1 ± 3.6	17.7 ± 5.5	0.786
Fat adiposity (%)			
Android	41.2 ± 9.4	46.2 ± 8.7	0.127
Gynoid	37.5 ± 7.9	43.5 ± 5.2	0.018*
Muscle mass (%)			
Arms	71.3 ± 10.2	62.5 ± 9.4	0.015*
Legs	69.9 ± 9.0	64.1 ± 6.3	0.045*
Appendicular	70.1 ± 8.9	63.8 ± 6.7	0.029*
Android	58.8 ± 9.4	53.8 ± 8.7	0.127
Gynoid	62.5 ± 7.9	56.5 ± 5.2	0.018*
Whole body	68.2 ± 8.5	61.3 ± 8.3	0.023*
Android/gynoid fat ratio	1.1 ± 0.3	1.1 ± 0.2	0.552
Body fat percentage (%)	32.0 ± 8.2	37.4 ± 7.8	0.062
ASMI	6.7 ± 1.0	6.5 ± 1.5	0.592
CS daily dose (mg/kg) (*n* = 21)	0.10 ± 0.11	0.08 ± 0.12	0.722
Duration of CS exposure (yr) (*n* = 21)	7.7 ± 5.9	5.8 ± 4.1	0.428
Immune medication used	5 (23.8)	5 (35.7)	0.474
Disease duration (yr)	11.9 ± 9.7	12.9 ± 12.1	0.773
Quality of life score	15.0 ± 11.7	13.6 ± 10.2	0.735
QMGS	10.3 ± 4.8	11.4 ± 5.6	0.543

MG, myasthenia gravis; QMGS, quantitative myasthenia gravis score; ASMI, appendicular skeletal muscle index. CS, corticosteroid. Data are presented as numbers (percentages) or mean ± standard deviation. * $p<0.05$

Table S4. Clinical features and body composition of MG according to disease duration

Variable	≤ 10 years (n = 17)	≥ 10 years (n = 18)	*p*-value
Male sex	8 (47.1)	5 (27.8)	0.305
Age (yr)	57.1 ± 8.8	55.2 ± 8.6	0.522
Age group (yr)			0.518
40–49	4 (23.5)	6 (33.3)	
50–59	4 (23.5)	6 (33.3)	
60–70	9 (52.9)	6 (33.3)	
Obesity	9 (52.9)	5 (27.8)	0.176
Sarcopenia	3 (17.6)	5 (27.8)	0.691
Body mass index (kg/m2)	25.8 ± 4.4	23.9 ± 4.6	0.204
Fat mass (kg)			
Arms	2.2 ± 0.9	1.9 ± 0.9	0.310
Legs	6.8 ± 3.1	6.7 ± 2.3	0.911
Appendicular	9.0 ± 3.7	8.6 ± 3.1	0.719
Muscle mass (kg)			
Arms	4.4 ± 1.3	4.2 ± 1.7	0.727
Legs	14.4 ± 2.6	12.9 ± 3.3	0.153
Appendicular	18.8 ± 3.7	17.1 ± 4.9	0.265
Fat adiposity (%)			
Android	46.6 ± 9.1	39.9 ± 8.5	0.032*
Gynoid	39.3 ± 7.8	40.5 ± 7.4	0.655
Muscle mass (%)			
Arms	66.0 ± 11.2	69.4 ± 10.2	0.352
Legs	69.0 ± 9.1	66.3 ± 7.8	0.344
Appendicular	68.3 ± 9.1	66.9 ± 8.2	0.646
Android	53.4 ± 9.1	60.1 ± 8.5	0.032*
Gynoid	60.7 ± 7.8	59.5 ± 7.4	0.655
Whole body	64.4 ± 8.3	66.4 ± 9.7	0.513
Android/gynoid fat ratio	1.2 ± 0.2	1.0 ± 0.2	0.007*
Body fat percentage (%)	35.6 ± 8.3	32.9 ± 8.5	0.344
ASMI	6.8 ± 1.0	6.5 ± 1.3	0.455
CS daily dose (mg/kg) (*n* = 21)	0.06 ± 0.08	0.12 ± 0.13	0.113
Duration of CS exposure (yr) (*n* = 21)	3.8 ± 2.4	8.9 ± 5.7	0.025
Quality of life score	15.6 ± 11.2	13.3 ± 11.0	0.551
QMGS	11.5 ± 5.4	10.1 ± 4.9	0.402

MG, myasthenia gravis; ASMI, appendicular skeletal muscle index; QMGS, quantitative myasthenia gravis score. CS, corticosteroid. Data are presented as numbers (percentages) or mean ± standard deviation. * $p<0.05$

Table S5. Clinical features and body composition of patients with MG according to steroid use

Variable	With steroids (n = 21)	Without steroids (n = 14)	*p*-value
Male sex	9 (42.9)	4 (28.6)	0.488
Age (yr)	57.1 ± 8.6	54.7 ± 8.7	0.430
Obesity	9 (42.9)	5 (35.7)	0.737
Sarcopenia	5 (23.8)	3 (21.4)	1.000
Body mass index (kg/m2)	24.2 ± 3.8	25.7 ± 5.6	0.343
Fat mass (kg)			
Arms	2.0 ± 0.9	2.2 ± 0.9	0.455
Legs	6.4 ± 2.5	7.1 ± 3.0	0.436
Appendicular	8.4 ± 3.2	9.3 ± 3.6	0.413
Muscle mass (kg)			
Arms	4.2 ± 1.3	4.3 ± 1.7	0.876
Legs	13.1 ± 2.9	14.4 ± 3.3	0.231
Appendicular	17.4 ± 4.2	18.7 ± 4.8	0.375
Fat adiposity (%)			
Android	43.0 ± 10.0	43.5 ± 8.6	0.885
Gynoid	39.7 ± 8.2	40.2 ± 6.7	0.861
Muscle mass (%)			
Arms	69.1 ± 10.7	65.8 ± 10.7	0.376
Legs	67.7 ± 8.7	67.5 ± 8.3	0.944
Appendicular	68.0 ± 9.0	67.1 ± 8.2	0.767
Android	57.0 ± 10.0	56.5 ± 8.6	0.885
Gynoid	60.3 ± 8.2	59.8 ± 6.7	0.861
Whole body	66.5 ± 9.7	63.8 ± 7.9	0.389
Android/gynoid fat ratio	1.1 ± 0.2	1.1 ± 0.3	0.935
Body fat percentage (%)	33.7 ± 9.4	34.9 ± 6.9	0.694
ASMI	6.6 ± 1.2	6.7 ± 1.3	0.713

MG, myasthenia gravis; ASMI, appendicular skeletal muscle index. Data are presented as numbers (percentages) or mean ± standard deviation

Table S6. Clinical features of MG who received steroids and sex- and age-matched controls

Variable	MG with steroids (n = 21)	Non-MG (n = 105)	Mean difference (95% CI) [a]	*p*-value
Body mass index (kg/m2)	24.2 ± 3.8	23.7 ± 3.4	0.49 (-1.10–2.08)	0.547
Fat mass (kg)				
Arms	2.0 ± 0.9	1.6 ± 0.6	0.31 (-0.10–0.72)	0.140
Legs	6.4 ± 2.5	5.6 ± 2.2	0.84 (-0.38–2.06)	0.177
Appendicular	8.4 ± 3.2	7.2 ± 2.7	1.15 (-0.43–2.73)	0.154
Muscle mass (kg)				
Arms	4.2 ± 1.3	4.4 ± 1.4	-0.16 (-0.53–0.22)	0.419
Legs	13.1 ± 2.9	13.3 ± 3.2	-0.15 (-0.95–0.66)	0.724
Appendicular	17.4 ± 4.2	17.7 ± 4.5	-0.30 (-1.45–0.84)	0.607
Fat adiposity (%)				
Android	43.0 ± 10.0	39.3 ± 8.6	3.71 (-0.46–7.89)	0.081
Gynoid	39.7 ± 8.2	37.7 ± 9.5	1.97 (-1.17–5.11)	0.219
Muscle mass (%)				
Arms	69.1 ± 10.7	72.0 ± 9.8	-2.90 (-6.90–1.10)	0.156
Legs	67.7 ± 8.7	70.3 ± 9.9	-2.60 (-6.05–0.86)	0.141
Appendicular	68.0 ± 9.0	70.7 ± 9.7	-2.71 (-6.17–0.76)	0.126
Android	57.0 ± 10.0	60.7 ± 8.6	-3.71 (-7.89–0.46)	0.081
Gynoid	60.3 ± 8.2	62.3 ± 9.5	-1.97 (-5.11–1.17)	0.219
Whole body	66.5 ± 9.7	68.8 ± 8.0	-2.27 (-6.23–1.68)	0.260
Android/gynoid fat ratio	1.10 ± 0.22	0.52 ± 0.17	0.58 (0.51–0.64)	<0.001*
Body fat percentage (%)	33.7 ± 9.4	30.0 ± 7.6	3.70 (-0.21–7.60)	0.064
ASMI	6.59 ± 1.16	6.63 ± 1.23	-0.04 (-0.32–0.25)	0.808

MG, myasthenia gravis; CI, confidence interval; ASMI, appendicular skeletal muscle index. [a] estimated using the generalized estimating equation. * $p<0.05$

Table S7. Body composition of MG not receive steroids and sex- and age-matched controls

Variable	MG without steroids (n = 14)	Non-MG (n = 70)	Mean difference (95% CI) [a]	*p*-value
Body mass index (kg/m2)	25.7 ± 5.6	23.9 ± 4.3	1.85 (-1.23–4.92)	0.239
Fat mass (kg)				
Arms	2.2 ± 0.9	1.7 ± 0.6	0.45 (0.03–0.87)	0.036*
Legs	7.1 ± 3.0	6.6 ± 3.4	0.54 (-1.08–2.17)	0.511
Appendicular	9.3 ± 3.6	8.3 ± 3.7	0.99 (-0.94–2.93)	0.315
Muscle mass (kg)				
Arms	4.3 ± 1.7	3.9 ± 1.0	0.38 (-0.25–1.02)	0.238
Legs	14.4 ± 3.3	12.5 ± 2.7	1.88 (0.84–2.93)	<0.001*
Appendicular	18.7 ± 4.8	16.5 ± 3.5	2.27 (0.82–3.72)	0.002*
Fat adiposity (%)				
Android	43.5 ± 8.6	41.6 ± 7.3	1.87 (-2.55–6.30)	0.407
Gynoid	40.2 ± 6.7	41.4 ± 8.5	-1.23 (-4.08–1.61)	0.396
Muscle mass (%)				
Arms	65.8 ± 10.7	68.8 ± 9.0	-3.03 (-7.46–1.40)	0.180
Legs	67.5 ± 8.3	66.2 ± 9.4	1.33 (-2.54–5.19)	0.501
Appendicular	67.1 ± 8.2	66.7 ± 9.1	0.36 (-3.30–4.01)	0.849
Android	56.5 ± 8.6	58.4 ± 7.3	-1.87 (-6.30–2.55)	0.407
Gynoid	59.8 ± 6.7	58.6 ± 8.5	1.23 (-1.61–4.08)	0.396
Whole body	63.8 ± 7.9	65.6 ± 7.2	-1.82 (-5.47–1.82)	0.326
Android/gynoid fat ratio	1.11 ± 0.27	0.50 ± 0.19	0.61 (0.51–0.70)	<0.001*
Body fat percentage (%)	34.9 ± 6.9	33.0 ± 6.9	1.83 (-1.70–5.36)	0.310
ASMI	6.75 ± 1.28	6.28 ± 0.96	0.47 (0.01–0.92)	0.043*

MG, myasthenia gravis; CI, confidence interval; ASMI, appendicular skeletal muscle index. Data are presented as mean ± standard deviation. [a] estimated using the generalized estimating equation. * $p<0.05$

Table S8. Multivariable linear regression for association of body composition and steroid use (with versus without)

Variable	Regression coefficient (95% CI)	*P* value
Body mass index (kg/m²)	-1.85 (-4.94, 1.25)	0.232
Fat mass (kg)		
Arms	-0.24 (-0.88, 0.40)	0.449
Legs	-0.43 (-2.29, 1.44)	0.642
Appendicular	-0.67 (-3.04, 1.70)	0.569
Muscle mass (kg)		
Arms	-0.38 (-1.07, 0.32)	0.274
Legs	-1.87 (-3.13, -0.62)	0.005*
Appendicular	-2.25 (-4.01, -0.49)	0.014*
Fat adiposity (%)		
Android	-1.04 (-7.59, 5.51)	0.748
Gynoid	1.18 (-2.80, 5.16)	0.548
Muscle mass (%)		
Arms	1.92 (-4.40, 8.25)	0.539
Legs	-1.54 (-6.40, 3.32)	0.523
Appendicular	-0.77 (-5.62, 4.09)	0.750
Android	1.04 (-5.51, 7.59)	0.748
Gynoid	-1.18 (-5.16, 2.80)	0.548
Whole body	1.83 (-4.12, 7.77)	0.535
Android/gynoid fat ratio	-0.07 (-0.19, 0.05)	0.235
Body fat percentage (%)	-0.54 (-6.28, 5.19)	0.848
ASMI	-0.38 (-0.88, 0.12)	0.131

CI, confidence interval; ASMI, appendicular skeletal muscle index;
Adjusted covariates for sex, age, disease duration and MGFA (type of MG).
* $p<0.05$
